# Recent Advances of Chitosan-Based Hydrogels for Skin-Wound Dressings

**DOI:** 10.3390/gels10030175

**Published:** 2024-02-29

**Authors:** Wei Guo, Xiaoyue Ding, Han Zhang, Zhenzhong Liu, Yanting Han, Qianqian Wei, Oseweuba Valentine Okoro, Amin Shavandi, Lei Nie

**Affiliations:** 1 College of Life Sciences, Xinyang Normal University, Xinyang 464000, China; weiguo@xynu.edu.cn (W.G.); dingxiaoyue123456@126.com (X.D.); zhanghan813822@163.com (H.Z.); hanyt@xynu.edu.cn (Y.H.); qianqian.wei@ulb.be (Q.W.); 2 Taizhou Key Laboratory of Medical Devices and Advanced Materials, Taizhou Institute of Zhejiang University, Taizhou 318000, China; 3 3BIO-BioMatter, École Polytechnique de Bruxelles, Université Libre de Bruxelles (ULB), Avenue F.D. Roosevelt, 50-CP 165/61, 1050 Brussels, Belgium; oseweubaokoro@gmail.com (O.V.O.); amin.shavandi@ulb.be (A.S.)

**Keywords:** chitosan, chitosan movdification, hydrogels, wound dressings

## Abstract

The management of wound healing represents a significant clinical challenge due to the complicated processes involved. Chitosan has remarkable properties that effectively prevent certain microorganisms from entering the body and positively influence both red blood cell aggregation and platelet adhesion and aggregation in the bloodstream, resulting in a favorable hemostatic outcome. In recent years, chitosan-based hydrogels have been widely used as wound dressings due to their biodegradability, biocompatibility, safety, non-toxicity, bioadhesiveness, and soft texture resembling the extracellular matrix. This article first summarizes an overview of the main chemical modifications of chitosan for wound dressings and then reviews the desired properties of chitosan-based hydrogel dressings. The applications of chitosan-based hydrogels in wound healing, including burn wounds, surgical wounds, infected wounds, and diabetic wounds are then discussed. Finally, future prospects for chitosan-based hydrogels as wound dressings are discussed. It is anticipated that this review will form a basis for the development of a range of chitosan-based hydrogel dressings for clinical treatment.

## 1. Introduction

As an important barrier to human defense, the skin plays a crucial role in protecting the body from mechanical damage, maintaining homeostasis, sensing external stimuli, and participating in immune responses [1,2,3]. However, the skin, as an outer barrier, is highly vulnerable to a range of external stressors. Wound healing is a complex, highly coordinated multi-stage dynamic biological process [4,5,6,7,8,9]. Additionally, the process of wound healing can be interfered with by numerous unknown factors such as infection, medications, wound size, genetics, immunosuppression, and radiation. The annual treatment of wound healing creates a huge social and economic burden around the world [10,11]. Nowadays, clinical wound management is still a major challenge.

The importance of wound dressings to promote wound healing has been known of for a long time. The dressing is a material that can be applied to the surface of a wound to create a clean environment and promote healing. As technological advancements progressed during the Industrial Revolution, people covered wounds with conventional materials, such as cotton, bandages, gauze, etc. However, these traditional dressings had limitations due to their poor wound-healing properties [12]. For example, these dressings exhibited weak antibacterial effects, which can lead to wound infections caused by bacteria or dust and are susceptible to causing inflammation. In addition, the frequent replacement of these dressings can easily lead to the reattachment of granulation tissue to the dressings, resulting in secondary injury to the wounds, which is not conducive to wound healing. More importantly, these traditional dry dressings failed to provide an effectively moist environment for wounds. Studies have shown that a moist environment is more conducive to cell proliferation, wound closure, and scarless healing. In order to achieve ideal healing results, it thus makes sense to develop wound dressings with advanced properties [13,14]. A range of new dressings are now available, including films, foams, composites, sprays, nanoparticles, and hydrogels, which act as barriers to promote wound repair [15,16,17].

Due to its unique properties, such as non-toxicity, biocompatibility, biodegradability, hydrophilicity, and soft texture, a hydrogel stands out as an excellent choice for wound dressings. Not only does it promote cell proliferation, but it also provides a moist wound-healing environment, making it the preferred option for wound dressings. Among the various hydrogels, chitosan-based hydrogels are considered suitable for hydrogel wound dressings due to their biodegradable, biocompatible, safe and non-toxic, bioadhesive, bacteriostatic, and hemostatic properties. At the same time, they can be loaded with therapeutic molecules or growth factors, enabling the regulation of drug delivery and release to promote faster and better wound healing [18,19]. This review, therefore, focuses on chitosan-based hydrogels for wound healing, highlighting their suitable properties and functions that make them desirable for different wound-healing applications.

## 2. The Wound Healing Process

**Figure 1:**
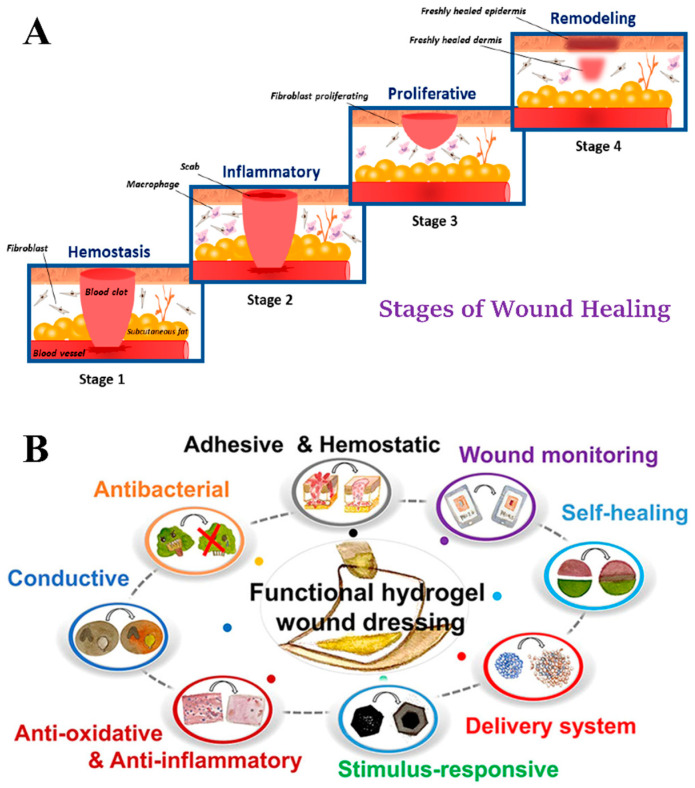
(**A**) The four stages of wound healing: hemostasis, inflammation, proliferation and remodeling [20]. (**B**) Various properties of multifunctional hydrogel wound dressings used for wound healing [26].

The wound healing process is complex, multi-stage, and dynamic. During this process, many types of cells, cytokines, growth hormones, and metal ions are highly coordinated and control the repair of the damaged tissue [4,5]. The process can be categorized into four phases: hemostasis, inflammation, proliferation, and remodeling (Figure 1A) [20]. When blood vessels are damaged, exposed basement membranes and tissue factors lead to platelet activation and aggregation. In addition, the production of thrombin prevents further bleeding [7,21]. During the inflammatory phase, injured cells release chemokines to attract immune cells such as macrophages and neutrophils to the injury site, the main task of which is to prevent and treat wound infections. Neutrophils have a strong phagocytosis and bactericidal function; they are capable of eliminating bacteria and necrotic tissue by the production of reactive oxygen species (ROS), antimicrobial peptides, and a variety of proteases [21]. In addition, macrophages release a plethora of growth factors (GFs) and cell signaling molecules, such as platelet-derived growth factor (PDGF), transforming growing factor-β1 (TGF-β1), insulin-like growth factor 1 (IGF-1), vascular endothelial growth factor-α (VEGF-α), etc., which are involved in angiogenesis, tissue remodeling and other mechanisms directly or indirectly involved in the subsequent wound-healing regulation [22]. The third phase of wound healing, the proliferative phase, which can last days or weeks, is marked by crucial events, including angiogenesis, cell proliferation, collagen synthesis, granulation tissue formation, and epithelialization. The newborn cells and blood vessels form a pink-colored, soft tissue known as granulation tissue. Replacing the transient collagen-containing extracellular matrix (ECM) with novel substrates can promote the conversion of fibroblasts to myofibroblasts to support re-epithelialization, which triggers wound contraction [8]. During the remodeling phase, type III collagen is gradually replaced by type I collagen as the main component, making the wound tissue tougher, and the granulation tissue gradually forms scars that lack cells and blood vessels [9]. The wound repair process is complex and overlapping; in the initial stage of wound formation, M1 macrophages play a major role in promoting inflammation, while M2 macrophages gradually replace M1 macrophages in the later stage. The balance of the two different types of macrophages is an important factor in wound healing. Wang et al. designed a hydrogel loaded with exosomes from human adipose-derived mesenchymal stem cells, which can effectively promote the polarization of M2 macrophages and promote angiogenesis and tissue repair, due to the presence of exosomes [23]. In the process of wound healing, GFs act as important signaling molecules, coordinating a series of key events. This is because a single growth factor cannot adapt to the complex wound environment and the cost of multiple recombinant GF combination strategies is too high [24]. Zhang et al. designed a wound dressing containing platelet-rich plasma (PRP), and the acquired dual-network gel was endowed with enhanced machinery. PRP in the hydrogel is a blood-derived product, which can secrete a variety of GFs after activation and can be entangled with GFs to enable sustained release, further improving the clinical application effect. The hydrogel can promote re-epithelialization, upregulate GF expression, and accelerate angiogenesis and wound healing in rat and pig wound models [25].

## 3. Hydrogel Wound Dressings

To promote the complex wound-healing process, researchers have developed hydrogel dressings that are more effective than conventional dressings such as cotton and gauze. The most important feature of these hydrogel dressings is that they can maintain a moist environment for the wound and the surrounding skin, which promotes epidermal cell migration and angiogenesis and promotes wound healing. Hydrogel is a three-dimensional (3D) network structure formed by the chemical or physical combination of natural or synthetic polymers. The porous structure of the hydrogel also favors the absorption of wound exudates, the high water content helps to keep the wound site moist and is proven to be soothing and cooling, which relieves the patient’s pain, and its high breathability accelerates wound healing [27,28]. Hydrogels can be used effectively in the treatment of burns and ulcers by reducing scab formation and supporting the debridement of difficult wounds. In addition, hydrogel dressings are easier to remove and replace, as they do not leave fibers in the wound bed and do not cause secondary injuries. 

In recent years, there has been an increase in the development of multifunctional hydrogel dressings that can be modified with different materials and manufactured using different methods to meet diverse needs (Figure 1B). For wound infections, which are a common problem in injuries, the most effective clinical treatment is with antibiotics. However, the overuse of antibiotics has led to the emergence of drug-resistant bacteria, which poses a serious challenge. To address this problem, antibacterial hydrogels have proven to be an effective solution [29]. The microenvironment of the wound changes due to microbial infection. To monitor these changes in real time, many smart hydrogel dressings have been developed to dynamically monitor infected wounds, which could allow early warning and treatment of the infection [30]. Hemostasis, the first step in wound healing, can be facilitated by the use of polymers with hemostatic properties, making them the preferred choice for wound dressings. Chitosan, a natural cationic polysaccharide, has several excellent properties, such as good biocompatibility, antibacterial activity, and hemostatic capabilities. Therefore, it has been extensively studied in the field of hydrogel wound dressings [31]. In the subsequent sections, the properties and applications of chitosan-based hydrogel dressings are examined.

## 4. Chitosan and Chitosan Modification 

**Figure 2:**
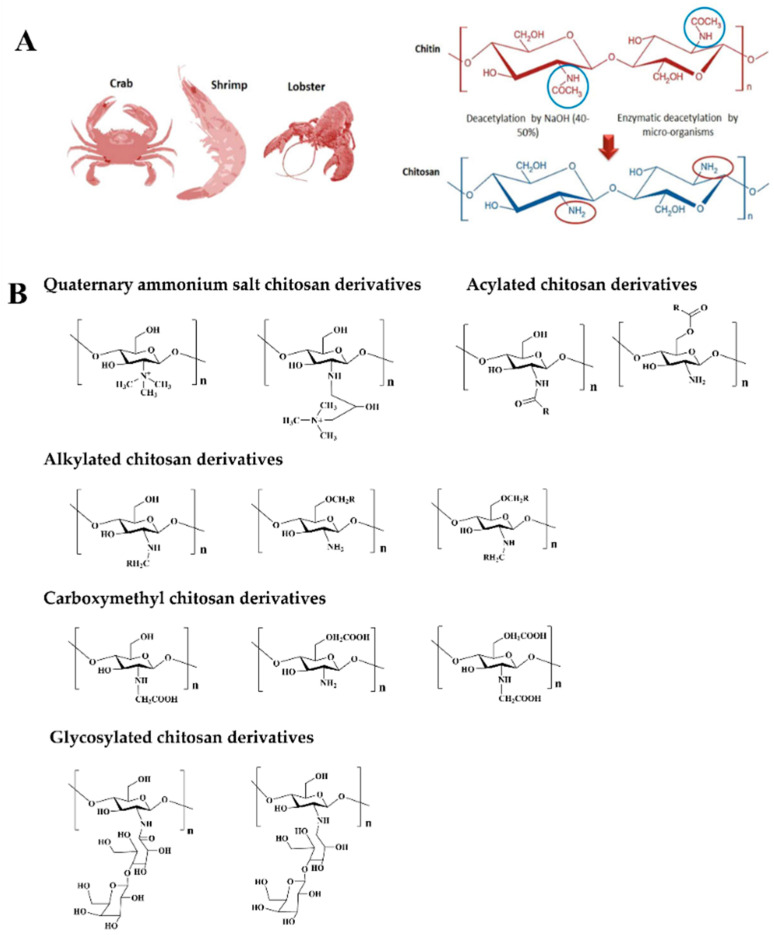
(**A**) Sources of chitosan [49]. (**B**) Different types of chitosan derivatives, including quaternary ammonium salt chitosan derivatives, acylated chitosan derivatives, alkylated chitosan derivatives, carboxymethyl chitosan derivatives, glycosylated chitosan derivatives, etc.

Chitosan is a rich natural basic cationic polysaccharide polymer consisting of N-acetylglucosamine and D-glucosamine monomer. It is an important derivative product obtained by the partial or complete deacetylation of chitin after long-term treatment in an alkaline solution [32]. Chitin is a common biopolymer in nature, second only to cellulose polysaccharides, and is mainly found in flora and fauna, together with insects, algae, crustaceans, and the cell walls of fungi [31]. It is formed by the N-acetylglucosamine molecule, linked by β-1,4-glycosidic bonds (Figure 2A) [33,34]. Chitin is limited in its use because it is poorly soluble in water. However, chitosan is soluble in acidic aqueous solutions due to its degree of acetylation. The physicochemical properties of chitosan are mainly affected by the degree of deacetylation (DD) and molecular weight (MW) [35,36,37].

In recent decades, chitosan has been widely used in various biomedical and biological applications, such as drug delivery, wound dressings, and tissue engineering, due to its unique biological activities such as biocompatibility, biodegradability, safety and non-toxicity, antibacterial activity, low immunogenicity, and antiviral properties [38,39,40]. In addition, chitosan also plays an important role in agriculture, industry, the food industry, and other fields [41,42]. The cationic functional groups on the chitosan chain can interact with the microbial cell wall and destroy microbial structure and function, thereby exerting antibacterial effects [43,44]. For example, Sun et al. investigated the preparation method for forming a complex from chitosan-copper gallic acid-based nanocomposites (CS-CU-GA NCs) with bifunctional nanoenzyme properties and antibacterial applications. They found that the complex exhibited excellent antibacterial activity and effectively killed *Staphylococcus aureus* and *Escherichia coli*. The antibacterial mechanism is such that its oxidase activity can generate hydrogen peroxide; the hydrogen peroxide is then catalyzed to generate hydroxyl radicals through peroxidase activity, thereby killing bacteria [45]. Chitosan also can provide a scaffold structure for cells growing in a targeted manner within the wound area. In addition, the functional groups of chitosan, such as amino groups and hydroxyl groups, can interact with receptors on the cell surface, promote the increase of cell adhesion and tissue adhesion, and show good biocompatibility [46,47]. For example, Xie et al. cross-linked carboxymethyl konjac glucomannan (CMKGM) and chitosan (CS) in different proportions to prepare CMKGM/CS composite sponges. The results showed that the sponge was not cytotoxic and did not cause the hemolysis of erythrocytes. Animal studies have shown that these sponges can promote the healing of skin wounds on the backs of mice and have the potential for use as wound dressings [48].

Despite its numerous advantages, chitosan’s inherent limitations, such as poor water solubility, weak mechanical properties, and poor thermal stability, often restrict its applications. Therefore, researchers commonly employ various modifications to enhance its functionality and expand the potential applications of chitosan. Common modification methods include both chemical and physical modifications. Chemical modifications (Figure 2B) can improve chitosan’s solubility, hydrophilicity, antibacterial properties, antioxidant properties, and other physicochemical characteristics, thereby gradually expanding its application range in areas such as wound dressings. Next, we will summarize the different chemical modifications of chitosan for the fabrication of hydrogel dressings, including quaternary ammonium-modified chitosan, acylated chitosan, alkylated chitosan, carboxymethyl chitosan, glycosylated chitosan, etc.

### 4.1. Chemical Modification

#### 4.1.1. Quaternary Ammonium-Modified Chitosan

Quaternary ammonium is an important modification of chitosan. Quaternary ammonium chitosan can be synthesized by directly introducing quaternary ammonium ions into the chitosan backbone or by reacting chitosan with quaternary ammonium. It has a variety of functions, such as strong antibacterial effect, good solubility, biocompatibility, biodegradability, non-toxicity, etc. Therefore, it is widely used as s flocculant, surfactant, and tissue culture material [50,51]. N,N,N-Trimethylchitosan (TMC) is the first quaternary chitosan ammonium derivative prepared using methyl iodide (MeI) as a methylating agent, with sodium iodide (NaI) as a catalyst and N-methyl-2-pyrrolidone (NMP) as a solvent, under alkaline conditions using the direct quaternary ammonium substitution method [49]. Hydroxypropyltrimonium chloride chitosan (HACC) is another widely used quaternary ammonium salt, which is formed by the reaction of chitosan and glycidyl trimethylammonium chloride and belongs to grafted quaternary ammonium [52].

#### 4.1.2. Alkylation-Modified Chitosan

Alkylated chitosan derivatives are produced by reacting chitosan with halogenated alkanes under alkaline conditions. In special cases, these can also be obtained by reacting chitosan with epoxy derivatives to yield N-alkylated chitosan derivatives. Due to the strong nucleophilic lone pairs of the amino groups of chitosan, N-alkylation products are more readily available while providing better protection of functional groups at specific sites [53,54,55]. The presence of alkyl groups breaks the hydrogen bonds of the chitosan molecules, so alkylated chitosan derivatives have better water solubility. In addition, it has been reported that N-alkylated chitosan has good biocompatibility and hemostatic activity [56]. Therefore, the hydrogels, including alkylated chitosan derivatives for use as the base material, are very promising as wound dressings. 

#### 4.1.3. Carboxymethylation Modification of Chitosan

Carboxymethylated chitosan (CMCS) comprises three main types: N-carboxymethylated chitosan (N-CMCS), O-carboxymethylated chitosan (O-CMCS), and N,O-carboxymethylated chitosan (N,O-CMCS). Compared to chitosan, carboxymethyl chitosan has better water solubility, biocompatibility, antibacterial activity, moisturizing properties, and antioxidant activity. Therefore, CMCS can be used for wound healing, medical skin care, bioengineering, etc. [57,58]. The carboxymethylation reaction mainly occurs at the C6-OH and C2-NH_2_ sites of chitosan, obtained by the treatment of chitosan with monochloroacetic acid and sodium hydroxide, and the nature of the products is affected by reaction conditions such as time, temperature, and solvent. For example, O-CMCS is available at room temperature, while higher temperatures are required for N-CMCS and N,O-CMCS [59]. The functional properties of CMCS are not only influenced by the molecular weight and the degree of acetylation of chitosan but also by the different types of CMCS derivatives. For example, substitution in N-CMCS occurs at NH_2_ so that the number of amino groups is reduced, resulting in the weaker antibacterial activity of N-CMCS than of O-CMCS [60].

#### 4.1.4. Acetylated Modified Chitosan

Acetylation modification refers to the introduction of aromatic or aliphatic acyl functional groups into the chitosan molecular chain, making it a common method of chitosan modification. Typically, organic acids or derivatives such as acid anhydrides and acyl chlorides are used as acylating agents. Acetylation reactions can be classified into N-acetylation and O-acetylation, based on the reaction sites [53]. Due to the different reactivity of hydroxyl groups in the chitosan molecule, the secondary hydroxyl group at the C3 position has high steric hindrance and limited rotational freedom, while the primary hydroxyl group at the C6 position has low steric hindrance and free rotation. The amine group in chitosan is more reactive than the hydroxyl group. Therefore, the acetylation order of chitosan is C2-NH_2_ > C6-OH > C3-OH. Researchers typically protect the amino group with substances such as benzaldehyde and methanesulfonic acid before removing the protective group through a reaction. This process ultimately yields O-acetylated chitosan [61]. The acetylation reaction interrupts the hydrogen bonds within and between the chitosan molecules and changes the crystal structure of chitosan. This modification improves the solubility, hydrophobicity, and lipophilicity of chitosan, thereby expanding its application possibilities and functionality in areas such as drug delivery systems, tissue engineering, and biomaterials [54].

#### 4.1.5. Phosphorylated Modification of Chitosan

The phosphorylated modification of chitosan involves introducing phosphate groups into the chitosan molecules, which improves the water solubility and metal-chelating properties of chitosan. This reaction is usually carried out at low temperatures, using phosphorus pentoxide as a starting material and methanesulfonic acid as a catalyst. This type of biopolymer has important applications in tissue regeneration, drug delivery, biofuels, and the food industry [31,32]. Additionally, research has also found that phosphorylated chitosan can be synthesized by grafting by reacting mono(2-methacryloyloxyethyl) phosphate with chitosan, which advantageously imparts amphiphilic properties to the chitosan and increases its antimicrobial activity [62,63].

#### 4.1.6. Graft Copolymerization

Chitosan has numerous active functional groups, and graft copolymerization can occur at the amino group (C2 position), the primary hydroxyl group (C6 position), and the secondary hydroxyl group (C3 position). Through graft copolymerization, various molecules or polymers can be linked to the main chain of chitosan, introducing multiple functional groups (such as alcohol, ester, acid, amide, etc.), which is an important research direction in the study of multifunctional chitosan. Common methods for graft copolymerization methods include oxidative coupling copolymerization, radiation grafting, radical graft copolymerization, enzyme graft copolymerization, etc. Chemical methods of graft copolymerization typically use initiators such as potassium persulfate, ammonium persulfate, iron sulfate, etc. [61,64,65]. This grafting copolymerization modification not only enhances the antibacterial, anticancer, and antioxidant abilities of chitosan polymers but also improves their solubility and biocompatibility [64].

#### 4.1.7. Other Chitosan Modifications 

In addition to the reactions mentioned above, commonly used chemical modifications of chitosan include glycosylation, sulfation, etherification, thiolation, etc. [62,63,64,65]. Research has shown that a chitosan Schiff base containing thiophene, indole, and pyrazole groups and CS was transformed into nanoparticles with high inhibitory activity on tumor cells [66]. Mohamed et al. developed a novel 1,3,4-thiadiazole-modified chitosan, which exhibited excellent antibacterial activity against *Escherichia coli*, *Staphylococcus aureus*, *Bacillus subtilis*, *Pseudomonas aeruginosa*, and *Candida albicans* [67]. Another study found that thiadiazole-modified chitosan possessed higher fungal inhibitory activity and the ability to kill early larvae compared to chitosan [68]. Chitosan can be chemically modified by clicking chemistry. Drozd et al. have synthesized a new n-methylimidazole-functionalized chitosan by copper-catalyzed azide-alkyne cycloaddition, enhancing its antibacterial activity and hemocompatibility [69].

Chitosan salts are also often used as biomaterials in various fields. In an earlier study, the researchers prepared a series of injectable, in situ-formed chitosan-based hydrogels that utilize ionic bond interactions between chitosan and sodium salts (trisodium phosphate (Na_3_PO_4_·12H_2_O), sodium sulfate (Na_2_SO_4_), sodium sulfite (Na_2_SO_3_), or sodium bicarbonate (NaHCO_3_) [70]. Four kinds of hydrogels were obtained by the chemical cross-linking of chitosan, sodium salt, and genipin, which can be used as a carrier of local curcumin, delay its release rate, and improve its bioavailability. Another study reported that a new non-toxic crosslinking agent, trisosodium 6-phosphogluconic trisodium salt, can be used to prepare hydrogels by ionic interactions with chitosan; these hydrogels are non-toxic, easy to extend, and can be attached to the skin as drug carriers for local administration or wound dressing without causing skin irritation [71].

#### 4.1.8. Physical Modification

The physical modification of chitosan is primarily about changing its properties through mechanisms such as charge interactions, entanglement, and crystallization. This can be achieved by physical cross-linking and mixing. Common physical cross-linking agents for chitosan include calcium chloride, TPP, and tannic acid. By blending, chitosan can take on the benefits of other materials, resulting in the better performance of chitosan composites. Common blending materials include drugs, nanoparticles, enzymes, and polymers [31,62].

## 5. Chitosan-Based Hydrogel Dressings

### 5.1. Quaternary Ammonium Chitosan-Based Hydrogels

Quaternary ammonium salt–chitosan-based hydrogels have been widely considered as wound dressings for their adorned antimicrobial properties. After the chitosan is modified using quaternary ammonium salts, quaternary ammonium chitosan-based hydrogels can be fabricated through Schiff base linkage, hydrogen bonding, and other mechanisms in conjunction with other compounds [72,73,74]. Compared to chitosan, the water solubility of quaternary ammonium chitosan is enhanced. In addition, the quaternary ammonium salt cations can further disrupt the bacterial cell membranes; thus, the antibacterial activities of the quaternary ammonium chitosan-based hydrogels were greatly enhanced [50]. 

**Figure 3:**
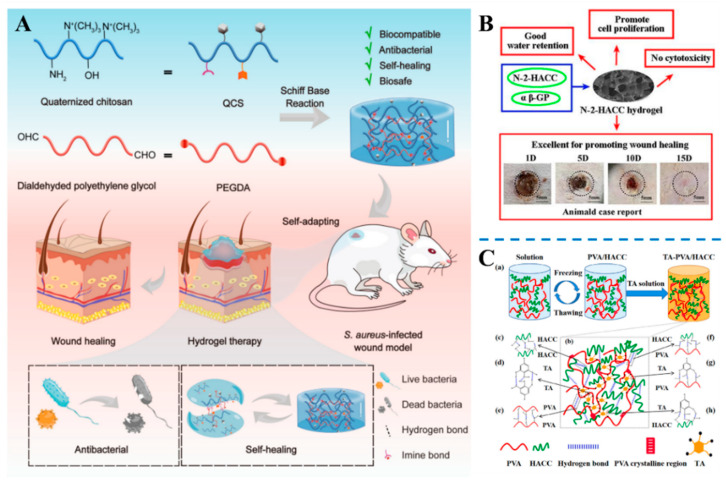
(**A**) The dynamic antibacterial hydrogel dressings prepared using quaternized chitosan (QCS) and dialdehyded polyethylene glycol (PEGDA) [75]. (**B**) Hydrogels prepared using N-2-HACC promote wound healing [76]. (**C**) A double-network hydrogel based on HACC, TA, and PVA was fabricated using a combination approach of cyclic freeze–thaw and soaking methods [77].

Cui et al. [75] have developed dynamic hydrogels by mixing quaternized chitosan (QCS) and dialdehydated polyethylene glycol (PEGDA); the mixture rapidly achieved the sol-gel transition through imine binding (Figure 3A). The prepared hydrogels exhibited self-healing and adaptive properties, and the irregular wound could be covered. In addition, the hydrogels possessed innate antimicrobial capacity. Thus, these hydrogels are promising as wound dressings against bacterial infections and are effective in closing wounds to control microbial infections. In addition, Wang et al. [76] also prepared an N-2-HACC hydrogel composite using N-2-hydroxypropyltrimethylammonium chloride-chitosan (N-2-HACC) as a raw material and αβ-sodium glycerophosphate (αβ-GP) as a crosslinker. The gelation of the obtained N-2-HACC hydrogel was short and stable at 37 °C, and the hydrogel exhibited excellent cytocompatibility and promoted cell proliferation. In addition, the hydrogel regulated the expression of growth factors such as TGF-β1, EGF, and VEGF, which could accelerate the formation of new blood vessels for wound healing (Figure 3B). Yao et al. [77] prepared a double network composite hydrogel by reacting 2-hydroxypropyltrimethylammonium chloride chitosan (HACC), tannic acid (TA), and polyvinyl alcohol (PVA) in a two-step process that facilitates the freeze–thaw cycle and immersion. The addition of tannic acid significantly improved the mechanical properties by forming a second layer of the network structure through hydrogen bonding. The composite hydrogel exhibited good antibacterial activities against both Gram-positive and Gram-negative bacteria, even without the addition of tannic acid. Cell experiments showed that the PVA/HACC/TA hydrogels exhibited good biocompatibility and also promoted the growth and proliferation of NIH 3T3 fibroblasts, confirming their potential for use as wound dressings (Figure 3C).

### 5.2. Alkylated Chitosan-Based Hydrogels

The introduction of a long alkyl chain into the chitosan can improve its hydrophobic properties, its solubility in organic solvents is further increased, and the hemostatic effect is greatly improved. Alkylated chitosan-based hydrogels are mainly used in hemostasis. The alkylated chitosan can be obtained by acylation, reductive alkylation, and so on [78]. Alkylated chitosan can be studied for its hemostatic properties by preparing it in various forms, such as sponges and hydrogels [79]. Compared to sponges, the porous structure of hydrogels allows the absorption of wound exudate and provides more space to collect plasma, which accelerates the clotting of blood cells at the wound site. Therefore, alkylated chitosan-based hydrogel dressings have significant advantages in hemostasis applications. Qiu et al. [80] prepared composite hydrogels from double-bonded modified N-dodecylchitosan (DCSG), polyethylene glycol diacrylate (PEGDA), and graphene oxide (GO). The prepared hydrogels showed excellent near-infrared assisted photothermal antibacterial properties against *E. coli* and *S. aureus*. In addition, they possessed good coagulation efficiency, which was confirmed by in vitro blood coagulation experiments. Palacio et al. [81] prepared alkylated chitosan and *N*-(3-chloro-2-hydroxypropyl) trimethylammonium chloride through free radical polymerization. Chen et al. [82] designed a series of N-alkylated chitosan (NACS) samples with different chain lengths and substitution degrees (SD) of alkyl groups. The alkylated chitosan-based hydrogels could rapidly interact with blood components such as red blood cells, platelets, and coagulation factors, leading to the accumulation of these blood components, the formation of a thrombus, and the activation of coagulation factors. The introduction of different carbon chain lengths and degrees of alkyl substitution can strongly influence the hemostatic ability of chitosan-based hydrogels. Therefore, alkylated chitosan-based hydrogels have great potential for wound dressings [79,82]. 

### 5.3. Carboxymethylated Chitosan Hydrogel

**Figure 4:**
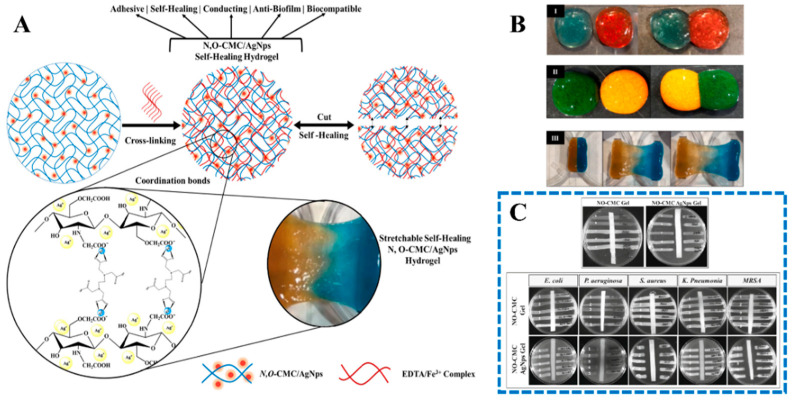
(**A**) N,O-carboxymethyl chitosan hydrogel with the incorporation of silver nanoparticles possesses adhesive, conductive, (**B**) self-healing, (**C**) antibacterial, and anti-biofilm properties [76].

Carboxymethylchitosan, also known as carboxymethylated chitosan (CMCS), has many beneficial properties, including good water solubility, cytocompatibility, antibacterial activity, and moisture binding. N,O-carboxymethyl chitosan (N,O-CMCS) offers improved hydrophilicity compared to chitosan. Pandian et al. [83] obtained self-healing, adherent, conductive, antibacterial, and antibiofilm hydrogels using adopted N, O-carboxymethyl chitosan, and silver nanoparticles using ethylenediaminetetraacetic acid-ferric ion (EDTA:Fe^3+^) complex (Figure 4). Chang et al. [84] reported multifunctional hydrogels based on heparin-modified N,O-CMCS and aldehyde-modified carboxymethylcellulose and encapsulated superoxide dismutase (SOD); the obtained hydrogel showed the ability to improve drug availability, reduce DNA damage, and shorten the inflammatory phase during diabetic wound healing. 

**Figure 5:**
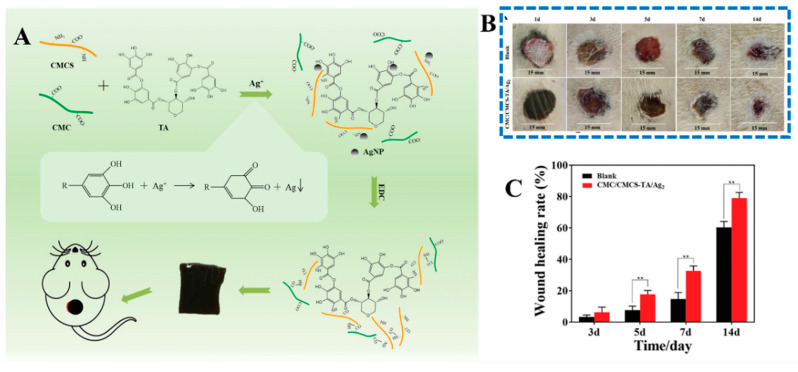
(**A**) Oxidized tannic acid-crosslinked carboxymethyl chitosan (CMCS) hydrogels incorporating silver nanoparticles exhibit good antibacterial properties and cytocompatibility, and the obtained hydrogel dressing could promote wound repair in a rat infection model (**B**,**C**) [78]. ** *p* < 0.01.

In addition, CMCS is commonly used as a cross-linking agent for the preparation of biomedical hydrogels due to its high amino and carboxy groups. For example, Xu et al. [85] have prepared a polysaccharide-based antibacterial hydrogel based on carboxymethylcellulose, CMCS, oxidized tannic acid, and silver nanoparticles using a synergistic combination interaction of EDC crosslinking and a Schiff base. The obtained hydrogels exhibited potential properties such as a low swelling rate, stable degradation rate, and excellent antibacterial activity. After the proper incorporation of silver nanoparticles, the hydrogel had no obvious cytotoxicity, and in vivo experiments confirmed that the hydrogel could promote the repair of the infected wound (Figure 5). Mo et al. [86] developed a carboxymethylcellulose-based hydrogel with double cross-linking, which was non-covalently cross-linked with Ca^2+^ and covalently cross-linked with 3-aminopropy-triethoxysilane (APTES). The designed hydrogel exhibited excellent cytocompatibility and hemocompatibility, and efficiently accelerated the healing of bacterially infected wounds. Recently, a number of papers have been published on hydrogels based on carboxymethyl chitosan interacting with other polymers such as oxidized sodium alginate [87], oxidized hyaluronic acid [88], γ-poly-glutamic acid [89], aldehyde-4-arm polyethylene glycol [90], etc. 

### 5.4. Acylated Chitosan-Based Hydrogels

Acylated chitosan also possesses excellent properties when used for hydrogel dressings. Khaleghi et al. [84] developed a honey-based double network hydrogel using polyvinyl alcohol (PVA) and acetylated chitosan. The first network of the hydrogel was formed by incorporating honey into a hydrogel obtained from PVA and acetylated chitosan, while the second network was formed through the reaction of maleic anhydride with chitosan. The flexibility of the obtained hydrogel could be optimized by adding a calcium chloride solution. The testing results demonstrated that the hydrogels exhibited good water absorption, mechanical strength, and biocompatibility. Zhou et al. [91] synthesized chitosan-based thiol-ene hydrogel using a click chemistry reaction between maleic acid-modified chitosan (MCS) and thiol-terminated poly (vinyl alcohol) (TPVA). The fabricated hydrogels displayed rapid gelation, along with good cell adhesion and proliferation abilities. Luo et al. [92] prepared a thermo-responsive hydrogel based on maleate chitosan and poly(N-isopropylacrylamide-*co*-maleic acid), with acetylated chitosan used as the crosslinking agent. The obtained hydrogels exhibited optimal properties as wound dressings for skin regeneration. Julia et al. [93] obtained N-acylated chitosan-based hydrogels using the microwave radiation method; the mechanical durability and stability were significantly improved compared to chitosan-based hydrogels. 

### 5.5. Phosphorylated Chitosan-Based Hydrogels

Phosphorylated chitosan (PC), a water-soluble derivative of chitosan, has many favorable properties in wound healing, such as hemostatic properties, anti-inflammatory, antioxidant, antibacterial, and angiogenic activity. Moreover, the behavior of cells such as fibroblasts and endothelial cells could be modulated by phosphorylated chitosan, due to the presence of anionic groups [94]. Liu et al. [95] designed a hydrogel using phosphocreatine salt, methacrylic acid (MA)-modified chitosan, and methacrylate gelatin (GelMA); this hydrogel could promote the osteogenic differentiation of osteoblast cells. Yang et al. [96] prepared a phosphorylated methacrylamide chitosan (PMAC) hydrogel based on methylacrylamide chitosan (MAC). Li et al. [97] obtained a polyelectrolyte complex (PEC) hydrogel using phosphorylated chitosan with chitosan acetate. The obtained hydrogels displayed excellent affinity with cells. 

### 5.6. Active Compound-Loaded Modified Chitosan-Based Hydrogels

Sustainable and controlled drug delivery is an important area of research. Chitosan-based hydrogels can be used as drug carriers, allowing drugs to pass more easily through biological barriers, achieving the ability to locally delay the delivery of various active drugs for the treatment of various clinical diseases [98]. Curcumin has much pharmaceutical potential but, due to its poor water solubility and bioavailability, it often fails to achieve the best therapeutic effect [99]. Li et al. embedded curcumin in a hydrogel formed from thiolated chitosan and liposome, which effectively prolonged the release of curcumin, significantly killed MCF-7 cells, and inhibited the recurrence of breast cancer [100]. Fibroblast growth factor 2 (FGF-2) is an important signaling molecule in wound healing. The researchers developed an FGF-2-incorporated CMCS/ hydroxyethyl cellulose hydrogel that significantly promoted skin tissue repair and regeneration and induced the expression of important active substances, including vascular endothelial growth factor A and matrix metalloproteinase 9 in repaired skin tissue, which can be used for burn treatment [101]. The porous hydrogel was used for the delivery and controlled release of FGF-2, to overcome the instability and short half-life of FGF itself and improve its bioavailability in the treatment of animal burns.

### 5.7. Other Chitosan-Based Hydrogels

Spizzirri et al. used chitosan as a raw material and, using the action of an ammonium persulfate initiator, synthesized thermosensitive hydrogels through a free radical-induced grafting process with N-isopropylacrylamide (NIPAAm). Gel synthesis was optimized by varying the amount of NIPAAm, and the characteristics of the hydrogel were studied using sodium 2,6-dichlorophenol as a model drug. The characterization data indicated that the hydrogel’s release rate of the drug was influenced by different temperatures [102]. Feng et al. prepared a hydrogel using chitosan–gallic acid graft copolymers, and the results demonstrated that the hydrogel exhibited excellent self-healing ability, adhesive properties, antioxidant performance, antimicrobial properties, hemostatic performance, and biocompatibility, making it a potential dressing for postoperative tissue repair [103].

Shikhani et al. grafted lactate onto chitosan and formed a physical crosslinking hydrogel. Fourier-transform infrared spectroscopy and X-ray diffraction confirmed the successful preparation of the hydrogel. In vitro and in vivo experiments demonstrated that the hydrogel exhibited excellent hemostatic ability, laying the foundation for the study of hemostatic dressings in wound healing processes [104]. Yi et al. mixed N-glycosylated chitosan and polyvinyl alcohol and loaded ofloxacin to prepare a series of multifunctional hydrogel dressings. In their in vivo wound model experiments, the hydrogel presented good antibacterial activity and great potential for promoting wound healing [105]. 

## 6. Properties of Chitosan-Based Hydrogel Dressings

### 6.1. Antibacterial Activity

**Figure 6:**
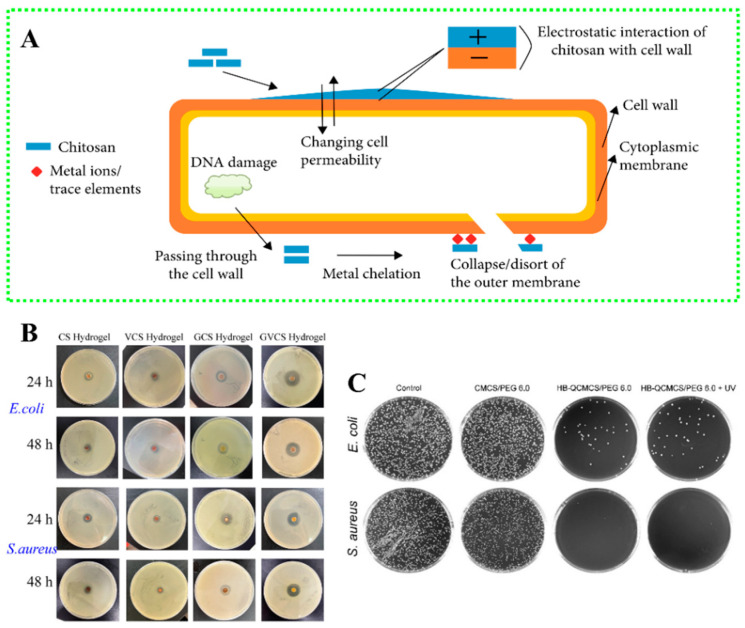
(**A**) Schematic diagram of the antibacterial mechanism of chitosan [106]; (**B**) the antibacterial results of a GVCS hydrogel [108]; (**C**) the HB-QCMCS/PEG hydrogel’s antibacterial results [109].

The antibacterial properties of chitosan are related to the degree of deacetylation, and its antibacterial mechanism is mainly manifested in destroying the cell structure of microorganisms, such as cell wall rupture and membrane perforation (Figure 6A) [106]. Wang et al. studied the reaction of chitosan and disaldehyde chitosan in gum hydrogel with a Schiff base, which showed excellent antibacterial activity via plate experiments [107]. Song et al. used glutaraldehyde as a crosslinking agent, a vanadium metal-organic polyhedron (VMOP-2), chitosan, and glucose oxidase (GOx) to prepare GVCS multifunctional hydrogels, which exhibited an excellent antibacterial effect on *Staphylococcus aureus* and *Escherichia coli*. In vivo experiments have shown that GVCS hydrogels can be applied to infected wounds (Figure 6B) [108]. In addition, chitosan derivatives, such as quaternary ammonium salt chitosan, carboxymethyl chitosan, etc., tend to exhibit better bacteriostatic activity due to related group changes. At the same time, the antibacterial activity of chitosan-based hydrogel dressings is improved. Wei et al. crosslinked photo-responsive polyethylene glycol (PEG) and carboxymethyl chitosan to form hydrogels via Schiff base bonds under ultraviolet irradiation conditions. This complex chitosan-based hydrogel possesses excellent antibacterial activity, antioxidant properties, good hemostasis, and cytocompatibility and can be used to support wound healing as a wound-healing dressing (Figure 6C) [109]. The antibacterial activity can be further and significantly enhanced by encapsulating drugs or adding antibacterial agents in chitosan-based hydrogels [110,111]. There is another work using the strategy of combining quaternary ammonium chitosan and silver nanoparticles using a one-pot method to develop antimicrobial hydrogel wound dressings that are more efficient and synergistic than chitosan derivatives alone [112].

### 6.2. Hemostasis

**Figure 7:**
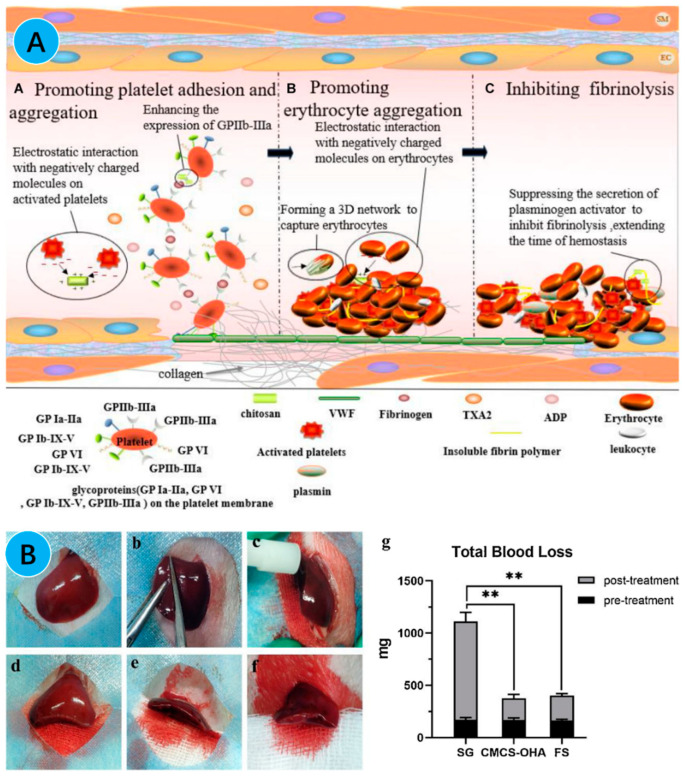
(**A**) Schematic diagram of the hemostatic mechanism of chitosan-based hydrogel [116]; (**B**) the hemostatic experiment and results using CMCS/OHA hydrogel [90]. ** p < 0.01.

Among the many candidates for hemostatic agents, chitosan stands out as a promising option. Its hemostatic mechanism is different from traditional coagulation factors. The hemostatic properties of chitosan mainly rely on its charge. The positively charged chitosan binds to the negative charge of the cell membrane surface, thereby promoting platelet adhesion and aggregation, erythrocyte aggregation, inhibiting fibrinolysis, and, finally, stopping bleeding. During the process, platelet adhesion and aggregation are key to hemostasis (Figure 7A). It relies on glycoprotein on the platelet membrane for signal activation and conduction and then works with collagen and fibrinogen to agglutinate the platelets into thrombi. In addition, a small amount of negative substance present on the surface of activated platelets can also bind to chitosan and promote platelet aggregation [111,112,113,114]. 

At present, the hemostatic properties of chitosan-based hydrogels are being widely studied in wound repair. For example, Song et al. successfully prepared DCS-PEGSH hydrogels from 3-(3,4-dihydroxyphenyl) propionic acid-modified chitosan (DCS) and sebacateic acid crosslinked-p-hydroxybenzaldehyde (PEGSH) modified with capping polyethylene glycol. The hydrogel could coagulate blood into clumps in the in vivo hemolysis experiments and could adhere to red blood cells. These results further proved that the chitosan-based hydrogel had hemostatic properties (Figure 7A) [115]. Xia et al. synthesized a multifunctional hydrogel, named CMCS-OHA hydrogel, by crosslinking with oxidized hyaluronic acid (OHA) and carboxymethyl chitosan (CMCS), based on the characteristics of chitosan and hyaluronic acid, respectively. They verified the hemostatic performance of hydrogels in an SD rat liver resection model. It was found that when treated with CMCS-OHA hydrogel, the amount of blood loss at the site of a liver injury was significantly lower than that in the control group, presenting good hemostatic ability (Figure 7B) [88].

### 6.3. Conductivity

Chitosan, a polysaccharide, has almost no electricity on its own. However, the development of chitosan-based hydrogels with conductive properties is expected to provide excellent natural materials for connecting biology and electronics, showing great potential in the field of bioelectronics and wound healing [116]. Chitosan-based hydrogels can be given conductive properties by modifying the chitosan, adding conductive polymers or agents, and modifying other compounds in the hydrogel. Common conductive polymers are frequently utilized, including poly (3,4-ethylenedithiophene) (PEDOT), polyaniline (PANI), polycarbazole (PZ), polythiophene (PT), polyacrylamide (PAN), and so on. Additionally, common conductive agents are carbon nanotubes (CNTs) and metal nanoparticles such as silver (Ag) and copper (Cu) [117]. 

Wu et al. divided chitosan-based hydrogels with conductivity into two types: conductive material-conjugated hydrogels and ionic conductive hydrogels, grouped according to the perspective of conductivity [118]. Li et al. prepared ionic conductive hydrogels (PAM@CS/TA-Fe) by the in situ polymerization of acrylamide (AM), using chitosan as the base material, and introducing the reducing agent, tannin acid, and the oxidant Fe^3+^, which is beneficial for forming a redox reaction to accelerate the polymerization speed. After connecting the PAM@CS/TA-Fe hydrogel and the LED bulb to form a complete circuit, the bulb shone, and its brightness gradually brightened as the tension increased, which showed a functional relationship between resistance and strain. Therefore, the hydrogel exhibited not only good conductivity but also potential as an ideal strain sensor [119]. Cong et al. prepared multifunctional conductive double-network hydrogels with excellent flexibility and strong tensile properties, using hydrogels that were dynamically crosslinked with chitosan and flexible polyacrylamide (PAAM) and doped with polyaniline [120].

### 6.4. Self-Healing Ability 

**Figure 8:**
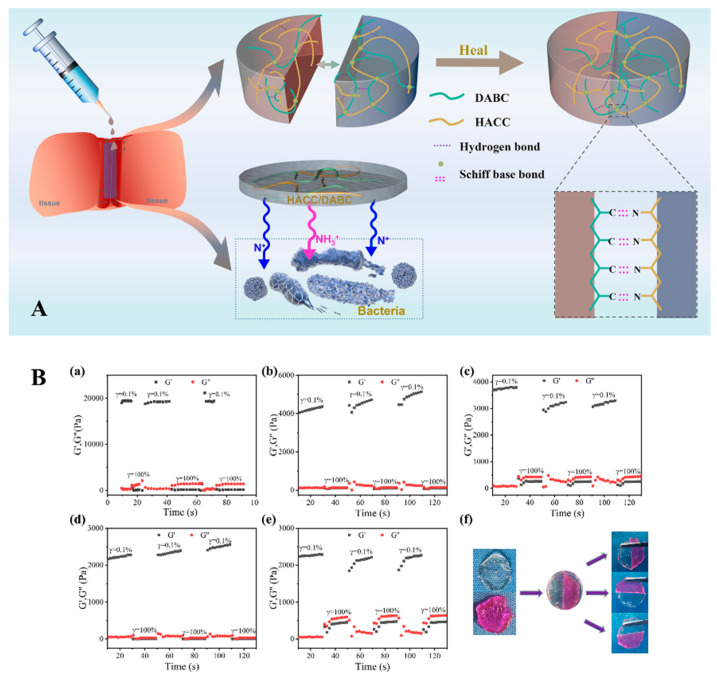
(**A**) Self-healing mechanism diagram of a chitosan-based hydrogel (HACC/DABC) [124]; (**B**) the self-healing properties of chitosan-based hydrogels (OQGG/CMCS) could be confirmed using Macro and micro evaluation methods [126].

The self-healing ability of hydrogel comes from the fact that, under appropriate conditions, the hydrogel can be recombined and reconnected after suffering external damage [121]. The groups on the chitosan molecular chain endow chitosan-based hydrogel with self-healing ability and antibacterial, hemostatic, and other properties [122,123]. The self-healing mechanism of hydrogel lies in forming a dynamic or reversible network structure via chemical covalent bonds (such as imine bonds, disulfide bonds, etc.) or physical non-covalent bonds (such as hydrogen bonds, hydrophobic bonds, etc.). These hydrogels with excellent self-healing properties also exhibit better persistence, responsiveness, and plasticity. Deng et al. prepared multifunctional chitosan-based hydrogels with self-healing ability via dynamic Schiff bases between amino groups in quaternary ammonium chitosan and aldehyde groups in dialdehyde bacterial cellulose (DABC) (Figure 8A) [124]. Li et al. used HACC and benzaldehyde-terminated polyethylene glycol (POSS-PEG-CHO) to produce a dynamic hydrogel network, and the resulting multifunctional hydrogel exhibited good self-healing properties [125]. Yu et al. used quaternary ammonium oxide guar gum (OQGG) and carboxymethyl chitosan to form a gel through the reaction of the Schiff base and prepared a hydrogel with self-healing, antibacterial, and other functionality (Figure 8B) [126].

### 6.5. Antioxidation

Reactive oxygen species (ROS) are a form of natural oxygen-containing chemicals that include superoxide anions (O^2−^), hydrogen peroxide (H_2_O_2_), hydroxyl radicals (HO^−^), and so on. ROS are mainly associated with the body’s metabolism processes. An excess level of ROS can lead to tissue damage and aging of the body and can even contribute to serious diseases. In addition, ROS is the main cause of chronic wounds. Therefore, there is an urgent and important need to investigate antioxidant hydrogel wound dressings [127]. 

Some chitosan-based hydrogels possess antioxidant properties, which can neutralize reactive oxygen radicals and reduce the damage caused by oxidative stress [128]. When combined with materials with excellent antioxidant properties, hydrogels with stronger ROS scavenging ability could be developed. Tannin, a naturally polyphenolic polymer with excellent antioxidant features, is commonly found in plants, especially nuts, fruits, tea leaves, and other plant parts. It is often used to prepare multifunctional hydrogel because of its variety of biological properties, such as antioxidant properties, anti-inflammatory effects, and anti-tumor effects. It is an ideal raw material for the preparation of antioxidant hydrogels [129]. For example, Azadikhah et al. used chitosan as the base material, tannin as an antioxidant, and N, N′-bis-(L-alanine)-3,4,9,10-peryltetramethylimide (PDI-Ala) as a photosensitizer to prepare a novel chitosan-based antioxidant photosensitizer hydrogel (CS-TA/PDI hydrogel) that exhibited great antioxidant properties [130]. 

### 6.6. Stimulus-Responsive Properties

The sensitivity of hydrogels to stimuli means that hydrogels can react to external influences such as pH value, temperature, concentration, light, electricity, etc. This unique property makes hydrogels highly versatile, finding a wide range of applications in drug delivery, biosensing, smart materials, and other fields. Some chitosan-based stimuli-responsive hydrogels showed different response characteristics, including two different types: the single-stimulus response and the multi-stimulus response [131]. Among these responsive hydrogels, pH- and temperature-stimulus-responsive hydrogels are common forms of stimulus-responsive hydrogels [132]. For example, Malik et al. designed a novel β-cyclodextrin-chitosan hydrogel with an adjustable pH response and investigated its role in drug release when the hydrogel was loaded with acyclovir [133]. Additionally, Yang et al. prepared supramolecular OACS-DAP hydrogels using orotic acid (OA)-modified chitosan (OACS) and 2,6-diaminourine (DAP). The rheological data showed that the hydrogel was temperature-dependent in the range from room temperature to 80 °C. OACS-DAP hydrogels also undergo different morphological changes between pH = 2 and pH = 7, thus demonstrating a dual response to the stimulation properties of temperature and pH [134].

## 7. Chitosan-Based Hydrogels for Skin-Wound Healing Applications

In this section, we provide an overview of the applications of chitosan-based hydrogels in the field of wound healing, including burn wounds, surgical wounds, infected wounds, and diabetic wounds, and briefly mention the preparation of various chitosan-based hydrogels and their associated functionalities. Although it is not possible to provide a comprehensive overview of all biomedical examples of chitosan-based hydrogel wound dressings, the studies discussed in this section are intended to emphasize the important and indispensable role of multifunctional chitosan-based hydrogels in various types of wound healing [135].

### 7.1. For Burn Wounds

A burn wound is the result of damage to the skin and tissues or organs by heat, including hot liquids, vapors, hot gases, flames, and red-hot metal liquids. Burns are the most severe types of wounds and can affect almost all organ systems, leading to significant mortality and morbidity. Standard clinical treatment like skin grafting is an effective way to treat early-stage burn wounds, which can improve the prognoses of patients with severe burns and reduce hospitalization and mortality [136].

However, the healing and treatment of burn wounds are relatively challenging, including the issues of skin damage, pain, slow wound healing, fluid loss, and bacterial infection. Therefore, the selection of appropriate materials for burn dressings is very important [137]. Studies have demonstrated that chitosan-based hydrogels can promote the healing of burn wounds. The overexpression of ROS in burned skin leads to chronic inflammation and hinders skin wound healing. To solve this problem, researchers prepared a hydrogel of carboxymethyl chitosan (CMCS) by crosslinking carboxymethyl chitosan and loading curcumin (Cur), a ROS-sensitive linker, to promote burn wound healing [138]. Among the CMCS hydrogel, carboxymethyl chitosan and curcumin play a key role in accelerating burn healing. Carboxymethyl chitosan, a special derivative of chitosan, is synthesized by replacing one or both of the amino (NH_2_) and hydroxyl (OH) groups in the glucosamine unit with a carboxymethyl (-CH_2_COOH) substituent [139], possessing high viscosity, low toxicity, and good biocompatibility [59]. However, these properties are not sufficient for use on burn wounds, as some anti-inflammatory and antioxidant drugs can achieve a better therapeutic effect. Curcumin, which is extracted from turmeric rhizomes, is a natural polyphenol with anti-inflammatory and antioxidant properties. It can not only scavenge excess reactive oxygen species but also reduce the cellular expression of pro-inflammatory cytokines (IL-6 and TNF-α) [140]. Using curcumin-loaded carboxymethyl chitosan hydrogel in complex burn wound microenvironments is beneficial for removing excess ROS. In addition, as the degradation behavior progresses, the loaded curcumin is released from the interior of the hydrogel to further remove ROS and inhibit inflammation, finally promoting wound healing. 

### 7.2. For Surgical Wounds

Surgical wound infections and uncontrolled postoperative bleeding are common clinical reasons for death [141,142]. It is reported that the mortality of patients with surgical infections is twice that of uninfected patients [143], and surgical bleeding rates range from 10% to 35% [144]. Excessive bleeding can cause serious illness, such as hypotension, organ dysfunction, and even death. During certain surgical procedures, hemostatic mechanisms cannot achieve rapid and effective hemostasis [145]. Therefore, the development of effective hemostatic materials for surgical wounds is urgently needed, especially for irregularly shaped, incompressible visceral, high-pressure arterial and venous bleeding wounds.

**Figure 9:**
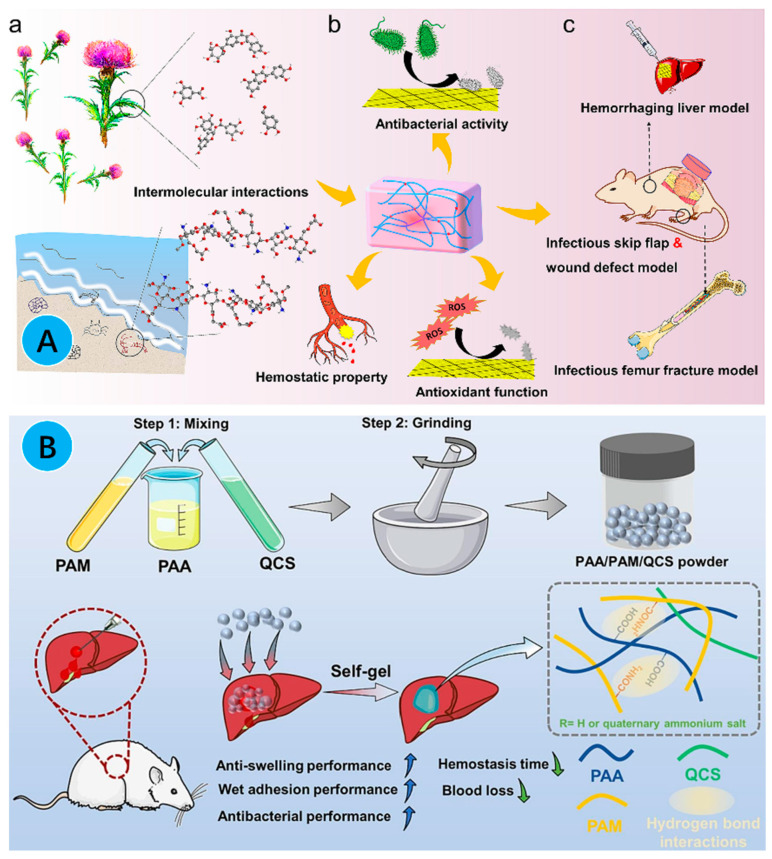
(**A**) Schematic representation of a CECS hydrogel, prepared with a mixture of carboxymethyl chitosan (CS) and traditional medicinal herb extracts (CE), which exhibits antibacterial, antioxidant and hemostatic activities in vitro and has shown good therapeutic effects in a model of hepatic hemorrhage, a model of skin flap infection, a model of an infected wound defect, and a model of infected femoral fracture [148]; (**B**) schematic diagram of the formation of PAA/PAM/QCS self-gelatinizing hemostatic powder and its application in hemostasis [149].

Biocompatible materials that incorporate multiple functions (e.g., antimicrobial and hemostatic properties) are ideal for wound dressings in surgery wounds. For example, *Cirsium setosum* has been used as an antihemorrhagic and anti-inflammatory agent for thousands of years [146,147]. The chitosan-based hydrogels showed the advantages of a good hemostatic effect, a good wound healing effect, easy application, low cost, good cytocompatibility, and good hemocompatibility for complex surgical wounds, which makes them a promising complementary biomaterial for the treatment of acute bleeding and the promotion of wound healing [145]. In view of these properties, the researchers reported a series of injectable and biocompatible multifunctional chitosan-based hydrogels containing cynomolgus extract (CECS hydrogel), a naturally derived compound, for the treatment of surgical wound infections and uncontrolled bleeding. The CECS hydrogels showed excellent hemostatic properties in the hemorrhagic liver in vivo (Figure 9A) [148]. In another study, quaternized chitosan (QCS) was shown to present good water absorption capacity, increased adhesion of blood cells and platelets, and antimicrobial properties that contribute to hemostasis. Therefore, the researchers reported an ultrafast self-gelling, wet-adhesive, and hemostatic polyethylenimine/poly (acrylic acid)/quaternized chitosan (PEI/PAA/QCS) hydrogel. The PEI/PAA/QCS hydrogel was applied to bleeding wounds and rapidly absorbed blood within 4 s, forming an adherent hydrogel in situ that could concentrate the clotting factors, maintain a stable, pressure-resistant physical barrier, attract blood cells and platelets, and improve hemostasis. At the same time, it has a rapid and effective hemostatic effect on irregularly shaped, incompressible organs (e.g., the liver and heart) and arterial hemorrhagic wounds, with high pressure in the rat model. In addition, the PEI/PAA/QCS hydrogel was effective in the hemostasis of incompressible organ hemorrhages with rich blood supply, such as porcine spleen and liver, and could promote the healing of full-thickness wounds (Figure 9B) [149]. 

### 7.3. For Infected Wounds

The disruption of skin integrity leads to wound formation. Once a wound occurs, the body regenerates the damaged or lost tissue through a complex set of repair mechanisms [150]. However, in some cases, the size and depth of the wound, infection, old age, and poor health can all affect the wound healing process and prolong healing time. Among these various reasons, infection is most likely to occur due to the presence of large amounts of nutrients at the wound site, leading to the failure of acute wound treatment and the development of non-healing wounds. Apart from hindering the healing process, wound infections can lead to serious complications such as osteomyelitis, sepsis, and even death [113]. 

The search for better antimicrobial strategies to avoid further damage from infection is always ongoing. In this section, the application of chitosan-based hydrogels in the treatment of infected wounds is presented. For example, Bai et al. prepared a GA-QCS/OHA hydrogel by grafting quaternary ammonium chitosan (QCS) with gallic acid (GA) and reacting it with oxidized hyaluronic acid (OHA). The hydrogel showed diverse properties such as injectability, hemostasis, degradation, and drug release. Among the compounds in the hydrogel, hyaluronic acid (HA) is a natural biomolecule. As a major component of the skin’s extracellular matrix (ECM), HA plays a role in inflammation, angiogenesis, and tissue regeneration. In addition, mupirocin is a topical antibiotic with strong antimicrobial activity against Gram-positive bacteria. A mupirocin-loaded multifunctional hydrogel based on QCS and HA showed significant antioxidant and migration-promoting effects in in vitro experiments. In addition, the bioactive effects of the hydrogel were tested using a full-thickness mouse wound model infected with *Staphylococcus aureus*. The results showed that the hydrogel inhibited the pro-inflammatory factor (TNF-α), upregulated the angiogenic factor (CD31), and accelerated the healing and regeneration of the infected wounds [151].

### 7.4. For Diabetic Wounds

Extensive data show that as of 2017, 30.3 million people have been diagnosed with diabetes in the United States alone [152]. Wounds in diabetics are more difficult to treat compared to wounds in non-diabetics. On the one hand, diabetic patients have a high blood glucose content in their tissue fluid, affecting tissue adhesion and making the wound site a favorable environment for breeding a large number of bacteria. On the other hand, prolonged inflammation, ischemia, and hypoxia are key factors that contribute to the failure of diabetic wounds to heal, likely leading to amputation unless treated appropriately. 

Hypoxia is a characteristic of diabetic wounds that can lead to the production of ROS; excess ROS disrupts the cellular redox balance, making the wound more difficult to heal [153,154]. In recent years, chitosan-based hydrogels with antioxidative capacity can provide a moist environment and further promote wound healing. In previous studies, the fluorinated methacrylamide chitosan (MACF) hydrogels showed that highly enhanced oxygen delivery [155,156] promoted collagen deposition during cellular processes and keratinocyte migration [157] and also improved wound healing [158] in splinting transgenic diabetic mouse wound models. Chitosan-based hydrogel presents a potential treatment for impaired diabetic wound healing [86,156].

Infection at the wound site impedes wound healing. In the clinic, systemic antibiotic therapy and conventional dressing care are not effective for chronic diabetic ulcers (DUs). Therefore, the researchers developed a series of sprayable antimicrobial hydrogels using QCS, with Mn as antimicrobial macromolecules, ε-polylysine-grafted graphene quantum dots (GQDs-ε-PL) as photothermal antimicrobial nanoparticles, and benzaldehyde-terminated tetra-armed poly (ethylene glycol) 4-armed PEG-BA for the therapy of DUs. The hydrogels react to the acidic environment induced by bacteria and exert a variety of antibacterial mechanisms, including photothermal antibacterial, chemical antibacterial and photodynamic antibacterial mechanisms. The synergistic antibacterial effect could kill bacteria more effectively, provide better healing conditions for wounds, and further promote the healing process of infected diabetic wounds [157]. In addition, researchers have developed a new injectable chitosan-based hydrogel that can load superoxide dismutase (SOD) and recombinant human epidermal growth factor (rhEGF) to improve drug utilization. This self-healing, degradable dual drug delivery system is formed by the modification of carboxymethyl chitosan with heparin and carboxymethylcellulose-formaldehyde, which can accelerate collagen fiber deposition and blood vessel formation, shorten the inflammatory period, and promote the healing of diabetic wounds [84]. The related chitosan-based hydrogels for different skin wound healing applications were briefly summarized in Table 1.

## 8. Conclusions

Recent research has shown that multifunctional chitosan-based hydrogel dressings can promote wound healing and facilitate wound treatment. In particular, these hydrogel dressings exhibit antibacterial, hemostatic, conductive, and other functions, and improve wound tissue repair by repelling microbial invasion and by promoting cell proliferation and migration. This review also highlights the use of chitosan-based hydrogel dressings for various types of wounds and demonstrates the value of these dressings. Moreover, it provides a solid foundation for further research and application of chitosan-based hydrogel dressings.

## 9. Future Outlook

This article investigates the chemical structures and properties of chitosan-based materials, as well as the function and application of chitosan-based hydrogels. The multifunctional chitosan-based hydrogel wound dressings showed good biocompatibility to promote cell proliferation and migration, excellent antibacterial activity against microbial invasion, and the effective promotion of wound healing. The use of chitosan-based hydrogels as wound dressings can, therefore, be used as a healing modality to achieve faster and better wound healing.

Although chitosan itself has excellent properties, a single component cannot fulfill multiple needs and functions. In the case of drug resistance caused by the misuse of antibiotics, the antimicrobial properties of chitosan alone are far from sufficient to solve this major problem. Therefore, in the production of chitosan-based hydrogel dressings, combinations involving photothermal or photodynamic antibacterial agents, metal nanoparticles, and antimicrobial peptides can be used to achieve an optimal antibacterial effect. The repair of chronic wounds is also a major clinical challenge, especially due to persistent inflammation. There are many studies dedicated to increasing the anti-inflammatory and antioxidant properties of chitosan-based hydrogels for the treatment of chronic wounds. Due to the important role that chitosan-based hydrogels play in wound healing, they are expected to further advance the development of wound healing techniques with advanced technology in the future. For example, exploring the intrinsic mechanisms of how chitosan-based hydrogel dressings promote the wound healing process is beneficial to further optimize the design strategies of advanced multifunctional chitosan-based hydrogels and meet the needs of clinical complex wound healing. In addition, many studies focus on promoting the wound healing process; the development of chitosan-based hydrogels that can perfectly repair scars and enable the skin to return to its pre-injury state is also the direction of future research.

Versatile chitosan-based hydrogels that have the ability to monitor and regulate the wound environment have attracted great attention from researchers. These hydrogels provide a better understanding of the wound healing process and real-time information on wound status. Changes in temperature, pH, uric acid, glucose, ROS, etc., at the wound sites can be detected and used for effective diagnosis to make early and accurate decisions for therapeutic interventions and add value to clinical medicine.

In addition, chitosan-based hydrogels using 3D printing technology and the combination of biology and electro-informatics could boost the trend toward smart wound management, enabling in situ wound monitoring and irregular wound coverage and healing. Delivering targeted treatments based on individual needs and monitoring wound healing data in real time can lead to more personalized treatment strategies.

## Figures and Tables

**Table 1:** Chitosan-based hydrogels for skin-wound healing applications.

Materials	Applications	Ref.
Thermosensitive poloxamer-chitosan-hyaluronic acid gel	Burn wound	[159]
*Cirsium setosum* extracts and carboxymethyl chitosan hydrogel	Surgical wound	[148]
Cefuroxime-conjugated chitosan hydrogel	Infected wound	[160]
Phellinus igniarius polysaccharide/chitosan-arginine hydrogel	Diabetic wound	[161]
Silver nanoparticle impregnated chitosan-polyethylene glycol hydrogel	Diabetic wound	[162]
